# Limited redundancy in genes regulated by Cyclin T2 and Cyclin T1

**DOI:** 10.1186/1756-0500-4-260

**Published:** 2011-07-26

**Authors:** Rajesh Ramakrishnan, Wendong Yu, Andrew P Rice

**Affiliations:** 1 Department of Molecular Virology & Microbiology, Baylor College of Medicine, Houston, TX 77030, USA; 2 Department of Pathology, University of Miami Miller School of Medicine/Jackson Memorial Hospital, Miami, FL 33136, USA

## Abstract

**Background:**

The elongation phase, like other steps of transcription by RNA Polymerase II, is subject to regulation. The positive transcription elongation factor b (P-TEFb) complex allows for the transition of mRNA synthesis to the productive elongation phase. P-TEFb contains Cdk9 (Cyclin-dependent kinase 9) as its catalytic subunit and is regulated by its Cyclin partners, Cyclin T1 and Cyclin T2. The HIV-1 Tat transactivator protein enhances viral gene expression by exclusively recruiting the Cdk9-Cyclin T1 P-TEFb complex to a RNA element in nascent viral transcripts called TAR. The expression patterns of Cyclin T1 and Cyclin T2 in primary monocytes and CD4^+ ^T cells suggests that Cyclin T2 may be generally involved in expression of constitutively expressed genes in quiescent cells, while Cyclin T1 may be involved in expression of genes up-regulated during macrophage differentiation, T cell activation, and conditions of increased metabolic activity To investigate this issue, we wished to identify the sets of genes whose levels are regulated by either Cyclin T2 or Cyclin T1.

**Findings:**

We used shRNA lentiviral vectors to stably deplete either Cyclin T2 or Cyclin T1 in HeLa cells. Total RNA extracted from these cells was subjected to cDNA microarray analysis. We found that 292 genes were down- regulated by depletion of Cyclin T2 and 631 genes were down-regulated by depletion of Cyclin T1 compared to cells transduced with a control lentivirus. Expression of 100 genes was commonly reduced in either knockdown. Additionally, 111 and 287 genes were up-regulated when either Cyclin T2 or Cyclin T1 was depleted, respectively, with 45 genes in common.

**Conclusions:**

These results suggest that there is limited redundancy in genes regulated by Cyclin T1 or Cyclin T2.

## Background

Positive transcription elongation factor b (P-TEFb) facilitates transition from abortive to productive mRNA elongation by phosphorylating the carboxyl terminal domain (CTD) of the large subunit of RNA Polymerase II (RNA Pol II) and also the negative elongation factors NELF and DSIF [1,2]. P-TEFb is essential for expression of most RNA Pol II-transcribed genes and P-TEFb function appears to be limiting for a large number of the non-expressed set of genes in different cell types [3,4]. P-TEFb exists in two forms in cells, a core P-TEFb and a snRNP complex. Core P-TEFb consists of Cdk9 as the catalytic subunit, a Cyclin subunit either Cyclin T1 T2 or K, and a protein known as Brd4 that is involved in directing core P-TEFb to active genes that are marked by acetylated histones [5]. The snRNP form of P-TEFb is catalytically inactive despite the presence of a Cyclin subunit and Cdk9 that is phosphorylated in its T-loop [6]. In addition to the core P-TEFb, the snRNP contains 7SK snRNA, HEXIM (either HEXIM1 or HEXIM2), MePCE (BCDIN3) and PIP7S (LARP7) proteins [5]. The precise function of the snRNP form of P-TEFb is unknown but it may serve to sequester excess Cdk9 and its Cyclin partner in a complex that can be readily recruited to activate RNA Pol II elongation [7].

The expression patterns of Cyclin T1 and Cyclin T2 differ in primary monocytes and CD4^+ ^T cells. In general, Cyclin T2 is expressed at a relatively high level in freshly isolated monocytes and its level remains constant when the cells are induced to undergo macrophage differentiation. In contrast, Cyclin T1 is expressed at low levels in monocytes and it is strongly up-regulated by a post-transcriptional mechanism when the cells are induced to differentiate to macrophages [8,9]. This up-regulation of Cyclin T1 protein expression appears to be required for the induction of a large portion of cellular mRNAs that are regulated during macrophage differentiation [10]. In resting primary CD4^+ ^T cells, Cyclin T2 levels are also relatively high and change little following T cell activation [11]. In contrast, Cyclin T1 levels are low in resting CD4^+ ^T cells and are strongly up-regulated following T cell activation by a post-transcriptional mechanism [11-13]. This expression pattern of Cyclin T2 and Cyclin T1 in quiescent vs. activated monocytes and CD4^+ ^T cells suggests that Cyclin T2 may be generally involved in expression of constitutively expressed genes in quiescent cells, while Cyclin T1 may be involved in expression of genes up-regulated during macrophage differentiation, T cell activation, and conditions of increased metabolic activity [14].

HIV-1 replication requires the viral Tat protein for productive RNA Pol II transcription of the integrated provirus. Tat functions by recruiting P-TEFb to the TAR RNA element that forms at the 5' end of nascent viral transcripts, where P-TEFb can phosphorylate the CTD, NELF, and DSIF. Tat makes direct protein-protein contact with Cyclin T1 and can therefore only utilize Cyclin T1-containing P-TEFb complexes. Inhibition of P-TEFb by siRNAs against Cyclin T1, a dominant negative-Cdk9 protein, or chemical inhibitors can inhibit HIV-1 replication *in vitro *[15-21]. It has been proposed that P-TEFb inhibitors have therapeutic potential for treatment of HIV-1 infection or cancer. A number of studies have also shown that P-TEFb inhibitors have potential as chemotherapeutic agents for some forms of cancer, such as chronic lymphocytic leukemia [22,23] or hepatocellular carcinoma [24]. A number of Cdk9 chemical inhibitors are currently being evaluated in clinical trials for treatment of various forms of cancer [25].

The effects of various P-TEFb inhibitors on cellular growth and cytotoxicity have been described in a number of studies. Stable expression of a dominant-negative Cdk9 protein has no observable effect on growth of a number of cell lines, although it does sensitize the monocytic U937 cell line to apoptosis [18,19]. Selicilib and flavopiridol, chemical inhibitors of Cdk9, can inhibit HIV-1 replication in cell lines at concentrations that are not cytotoxic [15,16]. Transient expression of siRNAs against Cyclin T1 or Cdk9 is able to inhibit HIV-1 replication without affecting the growth rate of HeLa cells [17]. However, stable depletion of Cdk9 using an shRNA vector that targets the 55 kDa isoform of Cdk9 induces apoptosis in HeLa cells [26]. A recent study generated a knock-out of the Cyclin T2 gene in mice and showed that Cyclin T2 is essential for vertebrate embryogenesis [27]. Transcriptional profiling in murine embryonic stem (ES) cells depleted for either Cyclin T2 or Cyclin T1 by transfected siRNAs identified a limited set of Cyclin T2- and Cyclin T1-dependent genes [27].

To further evaluate the effects of inhibition of P-TEFb function on cellular physiology, in this study we have used shRNA vectors to deplete Cyclin T1 and Cyclin T2 in HeLa cells where each of these P-TEFb subunits is expressed at relatively high levels. The shRNA vectors used here allow the stable depletion of target proteins, unlike previous studies that used transfections of siRNAs against Cyclin T1 which display only a transient depletion of the target proteins [17,27]. We found that stable depletion of either Cyclin T1 or Cyclin T2 had no effect on growth rates in HeLa cells. We carried out a transcriptional profile analysis in Cyclin T1- and Cyclin T2-depleted cells and identified cellular mRNAs whose expression is dependent upon Cyclin T1, Cyclin T2, or both Cyclin proteins.

## Findings

The expression patterns of Cyclin T1 and Cyclin T2 in primary monocytes and CD4^+ ^T cells suggests that Cyclin T2 may be generally involved in expression of constitutively expressed genes in quiescent cells, while Cyclin T1 may be involved in expression of genes up-regulated during macrophage differentiation, T cell activation and conditions of increased metabolic activity [9,11,12,14].

### Depletion of Cyclin T2 or Cyclin T1 in HeLa cells does not affect cell growth

We previously reported that the continuous expression of siRNAs against Cyclin T1 from a shRNA lentiviral vector displayed a stable knock-down and had no effect on the growth rate of either Jurkat CD4^+ ^T cells or MM6 monocytic cells [10,14]. We wished to compare the effects of stable depletions of Cyclin T1 and Cyclin T2, and therefore constructed shRNA vectors that target Cyclin T2. As a control shRNA vector, we used a shRNA lentiviral vector termed MM (mismatch) which contained a four nucleotide mismatch against the Cyclin T1 mRNA that has previously been shown to have only minimal effects on cellular mRNA expression levels [10]. The target sequence against Cyclin T2 was selected by a rational design strategy to a region common to both isoforms, Cyclin T2a and T2b [28].

**Figure 1:**
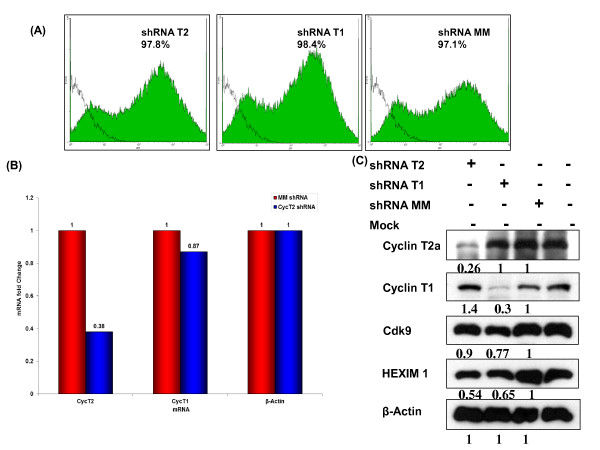
**shRNA against Cyclin T2 expressed from a lentiviral vector specifically and efficiently depletes CyclinT2 protein**. (A) HeLa cells infected at an m.o.i. of five with lentiviral vectors expressing a shRNA against Cyclin T2 (shRNA T2), Cyclin T1 (shRNA T1) or a control shRNA against a mismatch sequence in Cyclin T1 (shRNA MM). Cultures were analyzed five days post infection by flow cytometry. HeLa cells not treated with any shRNA were used as a control for flow cytometry analysis. The lentiviral vectors express a GFP marker protein. The unfilled region represents GFP background level in non-treated HeLa cells. The percentages of GFP positive cells are indicated. (B) Total RNA was extracted from HeLa cells transduced with shRNA T2 or shRNA MM after five days post infection and analyzed Cyclin T2, Cyclin T1 mRNA by quantitative real time RT-PCR. The fold-change is indicative of the transcript levels in the shRNA T2 treated cells relative to the shRNA MM treated cells after normalization to housekeeping gene, β-actin levels. (C) Immunoblot analysis of cell extracts prepared from HeLa cells transduced for five days with shRNA T2, shRNA T1 or shRNA MM lentiviral vectors. Untransduced HeLa cells (Mock) were used as control. The immunoblots were performed to analyze the levels of Cyclin T2, Cyclin T1, Cdk9, HEXIM1 and β-actin proteins. The band intensity was quantified using ImageJ and presented below each panel.

We first examined effects of Cyclin T2 and T1 depletions. HeLa cell cultures were transduced with lentiviral shRNA vectors against Cyclin T2, Cyclin T1 or the MM control and at five days post-transduction, flow cytometry analysis showed that > 97% of cells in all three cultures expressed the GFP marker protein (Figure 1A). We note that there are two populations of GFP^+ ^cells, high- and low-expressors. The explanation for these two populations is not known and we did not separate these two populations in the transcriptional profiling described below. The efficiency and specificity of Cyclin T2 depletion was examined by measuring the mRNA levels of Cyclin T2 and Cyclin T1 using quantitative real-time RT-PCR. The shRNA vector against Cyclin T2 reduced Cyclin T2 mRNA levels by ~ 60% relative to cells transduced with control vector, while Cyclin T1 mRNA levels was not significantly affected (Figure 1B). Two isoforms of Cyclin T2 are expressed in HeLa cells, termed Cyclin T2a and Cyclin T2b that contain different carboxyl termini due to differential splicing [29]. An immunoblot analysis showed that the protein level of Cyclin T2a was reduced when HeLa cells were transduced with the CyclinT2 shRNA vector but not Cyclin T1 shRNA vector (Figure 1C). Cyclin T2b protein is expressed at a very low level in HeLa cells and it was found to be reduced in cells transduced with Cyclin T2 shRNA vector but not Cyclin T1 shRNA vector (data not shown). The shRNA vector against Cyclin T1 was effective in depleting Cyclin T1 and had no effect on Cyclin T2a as we have observed previously [10]. We quantified the immunoblot relative to β-actin loading control and specific protein expression in cells transduced with MM control shRNA vector. We found Cyclin T2a and Cyclin T1 protein levels were reduced by ~74% and ~70% in HeLa cells transduced with CyclinT2 or CyclinT1 shRNA vector, respectively. We also observed that Cdk9 and HEXIM1 protein levels were reduced ~10 and 27% and ~46 and 35%, respectively, when either Cyclin T2 or Cyclin T1 was depleted. Other studies have reported similar findings [14,17,27] indicating that Cdk9 protein stability is linked to expression levels of its Cyclin partners. The reduction in levels of HEXIM1 could be the result of the stoichiometry of the 7SK RNP being disturbed when expression of Cyclin T2 or T1 and consequently Cdk9 are reduced.

**Figure 2:**
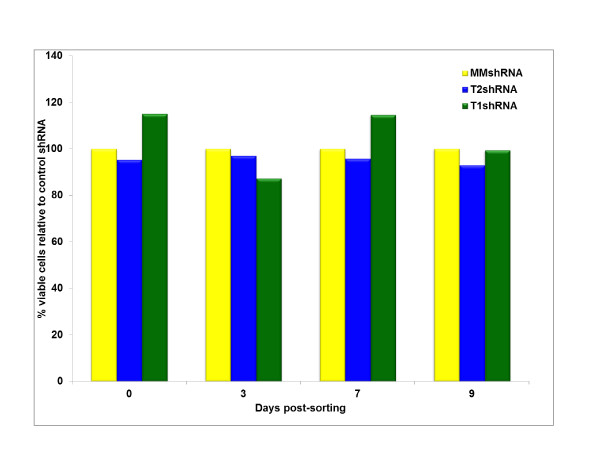
**Cyclin T2 depletion does not affect HeLa cell growth**. HeLa cells transduced with lentiviral shRNA vectors (shRNA T2, shRNA T1, shRNA MM) for 5 days were sorted by flow cytometry for GFP expression. The sorted (10^5 ^cells) cells were plated and MTT cell growth and viability assay was performed on days 0, 3, 7 and 9. Results are expressed as the percentage of viable cells compared with MM control shRNA transduced cells.

We examined the effect of Cyclin T2 and Cyclin T1 depletions on the growth rate of HeLa cells. Cultures were transduced with Cyclin T2 or Cyclin T1 shRNA vectors and five days later GFP positive cells in the cultures were sorted by flow cytometry. GFP positive cells were plated and MTT cell viability assays were carried out on the sorted cells at days 0, 3, 7 and 9. Cells expressing shRNA against Cyclin T2 or Cyclin T1 were not significantly affected in their viability or growth rate when compared with either mock or MM control transduced cells (Figure 2). It is possible that Cyclin T2 and Cyclin T1 may compensate each other in the activation of many genes. To investigate this, we attempted to concurrently deplete Cyclin T2 and Cyclin T1 but were not successful (data not shown). We previously observed that shRNA depletion of Cyclin T1 in monocytic MM6 cells had no observable effect on cellular viability or growth [10] while depletion of the 55 kDa isoform of Cdk9 in HeLa cells induced apoptosis [26]. We conclude from these data that the depletion of Cyclin T1 or Cyclin T2 under these conditions in HeLa cells does not affect cellular growth.

### Transcriptional profiling: validation and analysis of microarray data

The data presented in Figures 1 and 2 demonstrate that shRNA vectors against Cyclin T2 and Cyclin T1 are efficient and specific, and depletion of either protein in HeLa cells has no observable effect on cellular growth. This observation raises the question about the redundancy of these two Cyclin proteins for P-TEFb function. To address this issue, we carried out transcriptional profiling with DNA microarrays in cells depleted for Cyclin T2 or Cyclin T1. HeLa cells were transduced with shRNA-Cyclin T1, shRNA-Cyclin T2, or shRNA-MM Control lentiviral vector. At five days post transduction, cells were collected and total RNA was isolated. Additionally, a portion of cultures were monitored at this time for transduction efficiencies by flow cytometry using the vector GFP marker protein. Cultures were found to contain >97% GFP positive cells. Gene expression profiles were examined using the Affymetrix GeneChip Human Genome U133 PLUS 2.0 array, which contains about 54,000 probe sets representing approximately 18,953 unique (non-redundant) transcripts. Two independent biological replicate experiments were carried out in this analysis.

**Figure 3:**
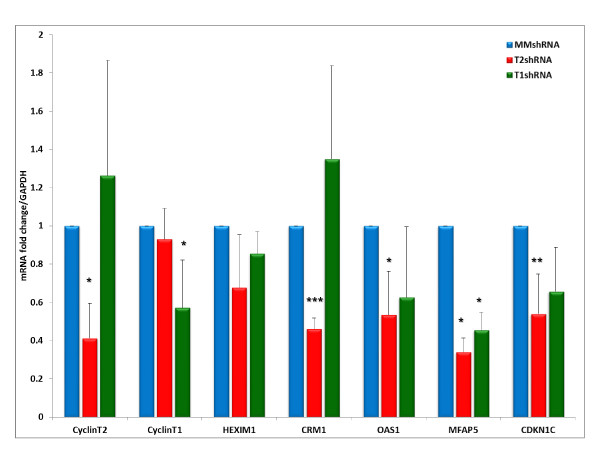
**Validation of microarray data**. Total RNA was extracted from four independent HeLa cell infections with indicated shRNA vectors for five days including aliquots of RNA for microarray analysis. Quantitative real-time RT-PCR was carried out to measure the expression level of indicated mRNA. The fold-change was calculated and represents the change in transcript levels in shRNA T2 or T1 infected cells relative to shRNA MM treated cells after normalization to housekeeping gene, GAPDH. Average fold-change from the four independent infections is presented. * p < 0.05, ** p < 0.005, *** p < 0.0005 in a paired t-test.

To assess the reliability of the microarray data, several mRNAs whose levels were differentially affected >1.2-fold by Cyclin T2 or Cyclin T1 depletions were selected for further analysis by quantitative real-time RT-PCR assays. These mRNAs were: Cyclin T2 (reduced 3.2-fold in DNA microarray data by Cyclin T2 depletion); Cyclin T1 (reduced 2-fold in DNA microarray data by Cyclin T1 depletion); HEXIM1 (reduced 2.5-fold in DNA microarray data by Cyclin T2 depletion); CRM1 (reduced 1.7-fold in DNA microarray data by Cyclin T2 depletion); OAS1 (reduced 2.5- and 2.4-fold in DNA microarray data by Cyclin T2 and Cyclin T1 depletions, respectively); MFAP5 (reduced 6.5- and 3.9-fold in DNA microarray data by Cyclin T2 and Cyclin T1 depletions, respectively); CDKN1C (reduced 2.2 and 2.6-fold in DNA microarray data by Cyclin T2 and Cyclin T1 depletions, respectively). RNA levels in real-time RT-PCR assays were normalized to GAPDH as the mRNA for this house-keeping gene was unaffected by Cyclin T2 or Cyclin T1 depletions. The transcript levels of the selected genes were analyzed from four independent biological replicate experiments. As shown in Figure 3, the changes in mRNA abundance in shRNA-Cyclin T2 and shRNA-Cyclin T1 treated cells relative to shRNA-MM control (set arbitrarily at 1.0) are in accordance with the trend seen in the microarray data. It should be noted that microarray data tend to be more compressed than that of quantitative real-time RT-PCR assays [30,31]. These data indicate that the microarray data are likely in general to be reliable.

### Cellular genes are differentially affected when either Cyclin T2 or Cyclin T1 are depleted in HeLa cells

We used the transcriptional profiling data from two independent biological experiments to identify the mRNAs that were either down-regulated or up-regulated > 1.2-fold (p-value < 0.05) by either Cyclin T2 or Cyclin T1 depletions relative to their expression in cells transduced with the MM control shRNA vector. The 1.2-fold cut-off has been used in other transcriptome analysis studies [32,33]. When a fold cut-off of 1.5 was examined, 96 total genes were down-regulated in Cyclin T2 depletions and 242 total genes were down-regulated in Cyclin T1 depletions. There were 75 and 147 genes up-regulated >1.5-fold when either Cyclin T2 or CyclinT1 was depleted, respectively. The intersection of Cyclin T2 and Cyclin T1 down-regulated genes >1.5-fold contained 35 total common genes, while the intersection of up-regulated genes contained 20 common genes (data not shown). When a fold cut-off of 2.0 was examined, 17 total genes were down-regulated in Cyclin T2 depletions and 71 total genes were down-regulated in Cyclin T1 depletions. There were 16 and 28 genes up-regulated >2.0-fold when either Cyclin T2 or CyclinT1 was depleted, respectively. The intersection of Cyclin T2 and Cyclin T1 down-regulated genes >2.0-fold contained just 5 total common genes, while the intersection of up-regulated genes contained 9 common genes (data not shown). It is likely that analyzing a high fold cut-off (>1.5- and >2.0-fold) will result in a gene list containing a number of false-negatives; conversely, analyzing a small fold cut-off (1.2-fold) will likely result in a gene list containing a number of false-positives. However, our analysis of the microarray data involved identification of differentially expressed genes using a student t-test and the Benjamini-Hochberg method was applied to correct for false discovery rate. The 1.2-fold cut-off was therefore chosen to include a wide representative of genes whose expression was changed by the shRNA treatment. Thus our gene lists are likely to contain relatively few false-negatives at the expense of containing some false-positives.

**Figure 4:**
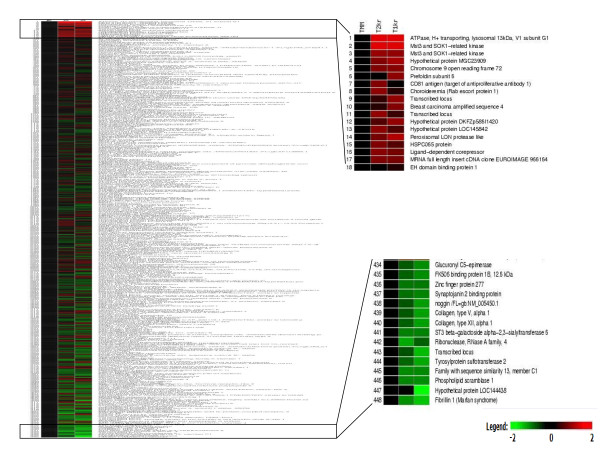
**Differential expression of genes following Cyclin T2 or Cyclin T1 knockdown**. Heat map of genes differentially expressed in HeLa cells infected with lentiviral shRNA vectors against Cyclin T2 or Cyclin T1 compared to control MM. Up regulated genes are in red and down regulated genes are in green. The insets represent an enlargement of a portion of the heat map.

As seen in the heat map in Figure 4, there are sets of genes that are either down-regulated or up-regulated in Cyclin T2 depletions, and different sets of genes that are down-regulated or up-regulated in Cyclin T1 depletions. The genes are arranged such that the up-regulated genes are at the top and the down-regulated genes at the bottom of the heat map. In addition, there are other sets of genes that are down-regulated or up-regulated in both Cyclin T2 and Cyclin T1 depletions.

**Figure 5:**
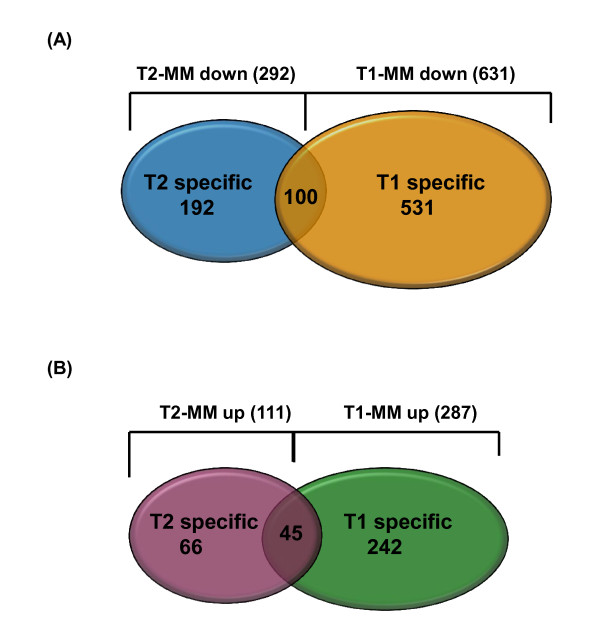
**Summary of pairwise comparisons for genes affected in shRNA T2 and shRNA T1 infected HeLa cells**. The Venn diagram represents the following individual pairwise comparisons: down regulated genes- shRNA T2 (blue circle) vs. shRNA T1 (orange circle), up regulated genes- shRNA T2 (purple circle) vs. shRNA T1 (green circle) each compared to shRNA MM. Number in parentheses represents the total number of genes whose expression was changed for that comparison. The numbers within each circle represents the genes unique to that comparison. The number in the intersection of the gene sets representing the shared genes whose expression was changed is indicated.

As shown in the Venn diagram in Figure 5, 292 total genes were down-regulated >1.2-fold when Cyclin T2 was depleted and 631 total genes were down-regulated >1.2-fold when Cyclin T1 was depleted. A total of 111 genes were up-regulated >1.2-fold in Cyclin T2 depletions and a total of 287 genes were up-regulated >1.2-fold in Cyclin T1 depletions (Figure 5B). The intersection of Cyclin T2 and Cyclin T1 down-regulated genes contains 100 total common genes, while the intersection of up-regulated genes contains 45 common genes.

### Redundant and non-redundant regulation of genes by Cyclin T2 or Cyclin T1 in HeLa cells

**Figure 6:**
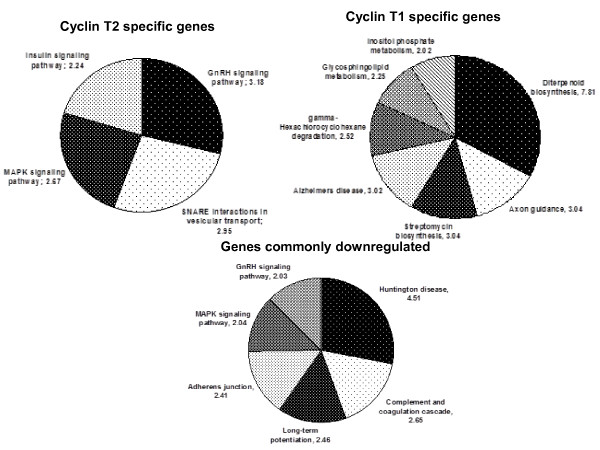
**KEGG pathway analysis of genes regulated by Cyclin T2 and Cyclin T1**. KEGG pathway grouping was done based on z-score following Gene Ontologic analysis of the unique and shared gene sets down regulated following HeLa cell infection with shRNA T2 or shRNA T1 lentiviral vectors compared to cells infected with control shRNA MM vector. The pie charts show over represented pathways for indicated conditions. The z-scores are also presented.

As expected, we identified genes that were up- or down-regulated by depletion of Cyclin T2 and Cyclin T1 (Figures 4, 5, 6). However, we were interested in characterizing the genes whose expression was down-regulated by the depletions, as these are more likely to be directly regulated by Cyclin T2 or Cyclin T1. The 10 genes showing the most down-regulation upon Cyclin T2 or Cyclin T1 depletion are shown in Table 1. Genes that were down-regulated only in Cyclin T2 depletions included Heat shock protein 3, HEXIM1 and Ankyrin repeat domain 12, while genes that were down-regulated only in Cyclin T1 depletions included Ankyrin repeat domain 29 and serine threonine kinase 38 (Table 1). Genes that were down-regulated in both Cyclin T2 and Cyclin T1 depletions include Forkhead box Q1 and Dual specificity phosphatase 16 (Table 2) indicating that genes important in immune response are under redundant control of both Cyclin T2 and T1.

**Table 1:** List of genes down regulated upon Cyclin T2 and Cyclin T1 depletion

Gene name	Gene ID	Gene identifier	Probe ID	Chromosome	Fold-change	p-value
**Top 10 genes down-regulated by Cyclin T2 depletion**						

**Heat shock 27 kDa protein 3**	HSPB3	NM_006308	206375_s_at	5	-3.12	0.0368

**G protein-coupled receptor 137B**	GPR137B	NM_003272	204137_at	1	-2.3	0.0251

**ATP-binding cassette, sub-family A (ABC1), member 1**	ABCA1	NM_005502	203504_s_at	9	-2.1	0.0336

**Ankyrin repeat domain 12**	ANKRD12	X80821	216563_at	18	-2.06	0.0166

**Ataxin 1**	ATXN1	AW235612	203231_s_at	6	-2.04	0.0111

**Coronin 6**	CORO6	NM_032854	1552301_a_at	17	-2.04	0.0330

**Growth arrest-specific 1**	GAS1	NM_002048	204457_s_at	9	-2.02	0.0303

**ATG16 autophagy related 16-like 1(S. cerevisiae)**	ATG16L1	NM_017974	220521_s_at	2	-1.95	0.0375

**Hexamethylene bis-acetamide inducible 1**	HEXIM1	NM_006460	202815_s_at	17	-1.94	0.0482

**Calcium channel, voltage-dependent, beta 2 subunit**	CACNB2	NM_000724	207776_s_at	10	-1.85	0.0178

**Top 10 genes down-regulated by Cyclin T1 depletion**						

**Chemokine (C-X-C motif) ligand 14**	CXCL14	NM_004887	218002_s_at	5	-3.55	0.0403

**Transmembrane protease, serine 3**	TMPRSS3	AB038160	223949_at	21	-2.59	0.0361

**Ankyrin repeat domain 29**	ANKRD29	AI307802	238332_at	18	-2.36	0.0433

**FK506 binding protein 1B, 12.6kDa**	FKBP1B	NM_004116	206857_s_at	2	-2.16	0.0108

**Syndecan binding protein (syntenin)**	SDCBP	NM_005625	203231_s_at	8	-2.09	0.0366

**DEAD (Asp-Glu-Ala-Asp) box polypeptide 18**	DDX18	NM_006773	205763_s_at	2	-1.9	0.0217

**Eukaryotic translation initiation factor 4E binding protein 2**	EIF4EBP2	U88989	224653_at	10	-1.89	0.0168

**Serine/threonine kinase 38**	STK38	NM_007271	1553117_a_at	6	-1.68	0.0196

**Cyclin G2**	CCNG2	AW134535	202769_at	4	-1.65	0.0129

**S100 calcium binding protein A4**	S100A4	NM_002961	203186_s_at	1	-1.62	0.0117

Genes with greatest down regulation specific to Cyclin T2 or Cyclin T1 depletion. The 10 genes with greatest decrease in expression following Cyclin T2 or Cyclin T1 depletion are listed (p 0.05). All fold-changes are relative to MM control. Minimum expression level of genes in MM control was arbitrarily chosen as 3.0.

**Table 2:** List of genes commonly down regulated by Cyclin T2 or Cyclin T1 depletion

Gene name	Gene ID	Gene identifier	Probe ID	Chromosome	Fold-change		p-value
					**Cyclin T2 depletion**	**Cyclin T1 depletion**	

**Aldo-keto reductase family 1, member C2**	AKR1C2	U05598	209699_x_at	10	-2.84	-2.23	0.0153

**Bone morphogenetic protein 2**	BMP2	AA583044	205289_at	20	-2.46	-2.18	0.0239

**Forkhead box Q1**	FOXQ1	AI676059	227475_at	6	-1.98	-2.18	0.0415

**Caspase 1, apoptosis-related cysteine peptidase (interleukin 1, beta, convertase)**	CASP1	U13699	211367_s_at	11	-1.95	-1.85	0.048914

**Dual specificity phosphatase 16**	DUSP16	AB051487	224832_at	12	-1.94	-1.32	0.0407

**Pre-B-cell leukemia transcription factor interacting protein 1**	PBXIP1	AI935162	214177_s_at	1	-1.90	-2.08	0.0185

**Bromodomain containing 9**	BRD9	NM_024786	1552283_s_at	5	-1.82	-1.7	0.007266

**Transcription factor 7-like 1 (T-cell specific, HMG-box)**	TCF7L1	NM_031283	221016_s_at	2	-1.81	-1.8	0.010119

**Peroxidasin homolog (Drosophila)**	PXDN	D86983	212013_at	2	-1.76	-1.83	0.0127

**1-acylglycerol-3-phosphate O-acyltransferase 3**	AGPAT3	BC004219	223184_s_at	21	-1.61	-1.79	0.01225

Genes commonly down regulated when either Cyclin T2 or Cyclin T1 is depleted. The 10 genes with greatest decrease in expression following Cyclin T2 or Cyclin T1 depletion are listed (p ≤ 0.05). All fold-changes are relative to MM control. Minimum expression level of genes in MM control was arbitrarily chosen as 3.0.

Gene Ontology and KEGG pathway analyses were carried out on the gene lists based on z-scores. The z-score is based on hypergeometric distribution and is calculated from the total number of genes in the array, the number of genes on the specific pathway, the number of genes on the gene list. Thus, a z-score of 0 indicates no enrichment, i.e., the expected number of hits is observed. A positive z-score indicates enrichment and a negative z score indicates under-representation.

Gene Ontology grouping showed that genes down-regulated by Cyclin T2 depletion included genes involved in response to stimulus and stress, negative regulation of metabolism and transcription, signal transduction and muscle development (Table 3). Genes down-regulated by Cyclin T1 depletion included genes involved in metabolism, intracellular signalling cascade, regulation of apoptosis, cell growth, transport, regulation of DNA repair, and antigen processing and presentation (Table 3). Genes commonly down-regulated by either Cyclin T2 or T1 depletion included genes involved in cellular processes, apoptosis, cell death, myeloid cell differentiation and lymphoid organ development (Table 3).

**Table 3:** Gene Ontology (GO) analysis of the gene list based on z-score

Ontology	List	Array	z-score
**Gene Ontologic analysis of genes down-regulated by Cyclin T2 depletion**			

**Biological process unknown**	14	566	3.38

**Response to chemical stimulus**	8	331	2.45

**Negative regulation of metabolism**	7	241	2.83

**Negative regulation of cellular metabolism**	6	204	2.65

**Negative regulation of transcription**	6	167	3.22

**Negative regulation of transcription, DNA-dependent**	6	109	4.56

**Enzyme linked receptor protein signaling pathway**	5	176	2.33

**Chromatin modification**	4	140	2.09

**Negative regulation of transcription from RNA polymerase II promoter**	4	66	3.98

**Regulation of kinase activity**	4	126	2.33

**Regulation of transferase activity**	4	128	2.3

**Regulation of Rho protein signal transduction**	3	69	2.68

**Regulation of small GTPase mediated signal transduction**	3	93	2.05

**Calcium mediated signaling**	2	22	3.69

**chloride transport**	2	43	2.31

**Energy reserve metabolism**	2	34	2.75

**Frizzled signaling pathway**	2	17	4.32

**Neurotransmitter transport**	2	40	2.44

**Oligosaccharide metabolism**	2	13	5.05

**Protein amino acid ADP-ribosylation**	2	24	3.49

**Protein processing**	2	46	2.18

**Ras protein signal transduction**	2	30	3.01

**Regulation of cyclin dependent protein kinase activity**	2	37	2.59

**Response to oxidative stress**	2	46	2.18

**Response to protein stimulus**	2	44	2.26

**Response to unfolded protein**	2	44	2.26

**Striated muscle development**	2	39	2.49

**Transmembrane receptor ptotein tyrosine phosphatase signaling pathway**	2	7	7.12

**Gene Ontologic analysis of genes down-regulated by Cyclin T1 depletion**			

**Metabolism**	178	6465	-2.45

**Cellular metabolism**	158	6076	-3.31

**nucleobase, nucleoside, nucleotide and nucleic acid metabolism**	64	2908	-3.24

**intracellular signaling cascade**	43	1038	2.05

**death**	28	555	2.72

**cell death**	27	551	2.51

**apoptosis**	26	524	2.53

**programmed cell death**	26	525	2.52

**regulation of apoptosis**	17	342	2.04

**Regulation of programmed cell death**	17	343	2.02

**small GTPase mediated signal transduction**	15	291	2.06

**Cellular morphogenesis**	14	247	2.36

**Regulation of enzyme activity**	13	235	2.18

**Protein folding**	11	197	2.04

**Anion transport**	10	160	2.32

**Growth**	10	174	2.04

**Cell growth**	9	139	2.32

**Endocytosis**	9	130	2.54

**Regulation of Cyclin dependent protein kinase activity**	4	37	2.72

**Antigen processing**	3	31	2.12

**RNA metabolism**	3	464	-3.11

**RNA processing**	3	383	-2.66

**post-Golgi vesicle mediated transport**	2	17	2.07

**Protein export from nucleus**	2	8	3.58

**Regulation of DNA repair**	2	7	3.89

**Antigen presentation, endogenous antigen**	2	16	2.17

**Antigen processing, endogenous antigen via MHC class I**	2	17	2.07

**Gene Ontologic analysis of genes down-regulated by Cyclin T2 or Cyclin T1 depletion**			

**Regulation of cellular process**	25	3058	2.04

**Regulation of cellular physiological process**	24	2840	2.17

**Negative regulation of biological process**	11	748	3.33

**Negative regulation of cellular process**	10	696	3.08

**Negative regulation of cellular physiological process**	9	631	2.89

**Negative regulation of physiological process**	9	655	2.77

**Positive regulation of biological process**	9	627	2.91

**Positive regulation of cellular process**	9	540	3.42

**Apoptosis**	8	524	2.93

**Cell death**	8	551	2.77

**Cell proliferation**	8	499	3.09

**Negative regulation of cell proliferation**	6	147	5.63

**Regulation of apoptosis**	6	342	2.91

**Innate immune response**	2	63	2.72

**Positive regulation of I-κB/NF-κB cascade**	2	76	2.37

**Myeloid cell differentiation**	2	37	3.88

**Hemopoietic or lymphoid organ development**	2	93	2.01

A GO analysis of the gene sets unique (192 and 531) and shared (100) to Cyclin T2 and Cyclin T1 depletion, respectively, was carried out and grouped based on z-score. Only GO categories that were over represented with at least 2 genes were considered. The "List" value is the number of affected genes from the gene list in the group. The "Array" value shows the total number of genes on the microarray that are in this gene ontology. The "z-score" is the expected number of genes in a GO term, subtracted from the observed number of genes. This value is divided by the standard deviation of the observed number of genes.

The gene list was also analyzed and grouped based on KEGG pathways (Figure 6). Genes affected by Cyclin T2 depletion were over-represented in signalling pathways (specifically MAPK, GnRH, and insulin signalling) and vesicular transport, while genes affected by Cyclin T1 depletion included genes involved in metabolism (lipid and inositol phosphate) and biosynthesis (diterpenoid and streptomycin). Interestingly, genes involved in MAPK and GnRH signalling pathways were over-represented in both Cyclin T2 and T1 depletions. While the over-represented pathways are similar, the identity of the genes are different suggesting that there is a limited degree of redundancy in the genes regulated by Cyclin T2 and Cyclin T1 even though there is a set of genes under specific control of either Cyclin T.

Dysregulation of specific genes could be a predisposition for development of a diseased state and because P-TEFb is responsible for transcriptional elongation of most protein coding genes [34], we next looked at the potential link between our gene list and diseases using the disease-gene link in DAVID http://david.abcc.ncifcrf.gov. As expected, the Cyclin T1 specific gene-list was over-represented for genes involved in HIV-1 infection. The other diseases implicated include dementia, cancers (colorectal and bladder), age related macular degeneration, cholesterol/LDL related and gallstones (Table 4). Cyclin T2 specific genes were over represented only in the aging process while metabolic and cardiovascular diseases were linked to genes that were commonly down-regulated upon Cyclin T2 or Cyclin T1 depletion (Table 4).

**Table 4:** Functional annotation of genes associated with disease

Condition	Disease/Processes	Genes
**Specific to Cyclin T2 depletion**	Aging	Fas (TNF receptor superfamily, member 6)
		
		N-acteyltransferase 1 (Arylamine N-acteyltransferase)
		
		ATP-binding cassette, sub-family A (ABC1), member 1
		
		Superoxide dismutase 2, mitochondrial
		
		Hemochromatosis

**Specific to Cyclin T1 depletion**	HIV-1 biology	Cyclin dependent kinase 9 (Cdk9)
		
		Cyclin T1
		
		Granulin
		
		Major histocompatibility complex, Class II, DM alpha
		
		Tubulin, beta 6
	
	Dementia	Amyloid beta (A4) precursor protein (peptidase Nexin-II, Alzheimer disease)
		
		Very low density lipoprotein receptor
		
		Estrogen receptor 1
		
		Microtubule-associated protein (Tau)
		
		Serpin peptidase inhibitor, clade 1 (Neuroserpin), member 1
		
		Synuclein, alpha (non A4 component of amyloid precursor)
	
	Cholesterol, LDL	Proprotein convertase Subtilisin/Kexin type 9
		
		Low density lipoprotein receptor (familial hypercholesterolemia)
		
		Apolipoprotein C-1
		
		Low density lipoprotein receptor adaptor protein 1
	
	Gallstones	Low density lipoprotein receptor-related protein associated Protein 1
		
		Low density lipoprotein receptor (familial hypercholesterolemia)
		
		Apolipoprotein C-1
	
	Macular degeneration, age-related	very low density lipoprotein receptor
		
		Microsomal Glutathione S-transferase 1
		
		Pleckstrin homology domain containing, family A (phosphoinositide binding specific) member 1
	
	Colorectal cancer	Platelet-derived growth factor receptor-like
		
		V-HA-Ras Harvey rat sarcoma viral oncogene homolog
		
		Mutated in colorectal cancers
	
	Bladder cancer	V-HA-Ras Harvey rat sarcoma viral oncogene homolog
		
		Fibroblast growth factor receptor 3 ( Achondroplasia, Thanatophoric dwarfism)

**Common to Cyclin T2 and Cyclin T1 depletion**	Metabolic	Fatty acid binding protein 3, muscle and heart (mammary-derived growth inhibitor)
		
		ATPase, class 1, Type 8B, member 1
		
		Heme oxygenase (decycling) 1
		
		Syndecan 2 (heparin sulfate proteoglycan 1, cell surface-associated, fibroglycan)
		
		Bone morphogenetic protein 2
		
		Calmodulin 1 (Phosphorylase kinase, delta)
		
		Hydatidiform mole associated and imprinted
		
		Scavenger receptor class B, member 1
		
		Smad, mothers against DPP homolog 4 (Drosophila)
		
	Metabolic	TIMP metallopeptidase inhibitor 3 (Sorsby fundus dystrophy, psudoinflammatory)
		
		Fibrillin 1 (Marfan syndrome
	
	Cardiovascular	Neural precursor cell expressed, developmentally downregulated 4-like
		
		Cyclin dependent kinase inhibitor 1C (p57, Kip2)
		
		Caspase 1, Apoptosis-related Cysteine peptidase (Interleukin 1, beta, convertase)
		
		Heme oxygenase (decycling) 1
		
		Calmodulin 1 (Phosphorylase kinase, delta)
		
		Poliovirus receptor-related 2 (Herpesvirus entry mediator B)
		
		Scavenger receptor class B, member 1
		
		TIMP metallopeptidase inhibitor 3 (Sorsby fundus dystrophy, psudoinflammatory)

The gene sets were investigated for potential link to diseases in a functional annotation analysis using DAVID. The disease states and the identity of genes potentially associated with it in the gene sets are presented.

## Discussion

In this study, we employed shRNA lentiviral vectors and cDNA microarrays to profile changes in mRNA expression levels in HeLa cells in response to long term depletion of the P-TEFb regulatory subunits Cyclin T2 and Cyclin T1. Using a 1.2-fold cut-off, we found that 292 and 631 genes were down-regulated upon depletion of Cyclin T2 and Cyclin T1, respectively. On the other hand, 111 and 287 genes were up-regulated upon depletion of Cyclin T2 or Cyclin T1, respectively. Interestingly, expression of 100 and 45 genes were commonly down-regulated or up-regulated, respectively, when either Cyclin was depleted. These data indicate that there is limited redundancy in the genes regulated in HeLa cells by either of the Cyclin partners of Cdk9 in P-TEFb complexes.

P-TEFb is required for the transcriptional elongation of most protein coding genes in mammals [34]. The P-TEFb complex containing Cyclin T1 has been examined in detail because it is an essential host factor in HIV-1 gene expression. However, Cyclin T2 has been reported to play a role in myocyte differentiation [35,36]. In this study, we found that depletion of either Cyclin T2 or Cyclin T1 under our conditions did not significantly affect cell growth or viability in HeLa cells. Thus, it is likely that a certain degree of redundancy is built into the genes regulated in transformed cells by either Cyclin T1and Cyclin T2. A recent report found that a Cyclin T2 knock-out resulted in embryonic lethality in mice [27]. In the same study, it was also reported that Cyclin T2 and T1 serve mostly redundant but also some non-redundant functions in murine ES cells [27]. In *C.elegans*, ablation of either Cyclin T2 or Cyclin T1 had no deleterious effect, indicating a high degree of redundancy in the functions of the Cyclins, although depletion of both Cyclins resulted in embryonic lethality [37]. Thus, there appears to be a degree of redundancy in the role of Cyclin T2 and T1 across species.

The expression of Cyclin T2 and T1 varies in primary hematopoietic cells. While Cyclin T2 is constitutively expressed in resting CD4^+ ^T-cells and monocytes, Cyclin T1 expression is very low in these cells [8,12]. Activation of CD4^+ ^T-cells and differentiation of monocytes into macrophages up-regulates Cyclin T1 levels by post-transcriptional mechanisms while Cyclin T2 level remains relatively constant [8,10,12,38,39]. We speculate that the expression pattern of both Cyclins reflect the nature of the genes that they regulate in these cells. We found that genes involved in metabolism (inositol phosphate and glycosphinglipid), biosysnthesis (Diterpenoid), and degradation (phenolics) were down-regulated upon Cyclin T1 and not Cyclin T2 depletion, whereas Kohoutek *et al. *[27] report that genes involved in cell cycle and communication were down-regulated. When Cyclin T2 was depleted, genes of signaling pathways (MAPK, GnRH and Insulin) and vesicular transport were down-regulated, while the Kohoutek study [27] found that the mRNAs of Notch, Wnt and TGFβ signaling and autophagy-related genes were affected. These differences in the signaling pathways whose expression specifically changes upon Cyclin T2 depletion is likely due to the different experimental system used-- the Kohoutek study used siRNA depletions in mouse ES cells, while we used shRNA depletions in HeLa cells [27]. We found that genes involved in signaling, cell junction, long term potentiation of nerve synapse and Huntington's disease were commonly down-regulated when either Cyclin was depleted. This suggests that Cyclin partners (both T2 and T1) of Cdk9 play a role in the nervous system and underscores the importance of P-TEFb in key cellular processes.

In most cell types examined so far, it appears that Cyclin T1 is the major regulatory subunit in P-TEFb complexes, while Cyclin T2a and T2b are minor regulatory subunits. This suggests that more genes are regulated by Cyclin T1 than T2. In agreement with this notion, we found that in HeLa cells there were 631 genes repressed by Cyclin T1 depletion, while 292 genes were repressed by Cyclin T2 depletion. In contrast, Kohoutek *et al. *found that in mouse ES cells, 59 and 76 genes were affected when either Cyclin T1 or Cyclin T2 were depleted, respectively [27]. We had reported earlier that Cyclin T1 depletion repressed 644 genes in PMA+ionomycin activated Jurkat cells, 965 genes in PMA-treated MM6 monocytic cells, and 778 genes repressed in LPS- treated MM6 cells [14]. It therefore appears that more genes are under the control of Cyclin T1 than Cyclin T2, with the exception of mouse ES cells and perhaps other select primary cell types. While P-TEFb is involved in expression of most protein coding genes, the number of genes affected by the depletion of Cyclin T2 or Cyclin T1 in our and the Kohoutek *et al. *study [27] is not very high. We speculate that there could be a subset of genes having a low threshold requirement for functional P-TEFb which are not affected by the reduction in Cyclin T2 or Cyclin T1 expression following shRNA depletions. These genes may be affected if Cyclin T2 or Cyclin T1 is genetically ablated. It is also possible that a certain degree of redundancy exists for genes regulated by Cyclin T2 and Cyclin T1 as was seen here and by Kohoutek *et al.*[27]. In addition to Cyclin T2 and Cyclin T1, Cdk9 has been reported to associate with Cyclin K [40,41]. While we have not examined the role of Cyclin K in this study, it is possible that when Cyclin T2 or Cyclin T1 is depleted, the Cdk9-CyclinK complex might provide an additional level of redundancy to the regulation of genes by P-TEFb.

In our study, genes involved in negative regulation of transcription and metabolism were down-regulated upon Cyclin T2 depletion, while genes involved in cell growth, metabolism, apoptosis, cellular morphogenesis and signaling were down-regulated upon Cyclin T1 depletion. Genes that were commonly down-regulated when either Cyclin T2 or T1 was depleted included the Gene Ontologic class of positive and negative regulation of cellular processes, apoptosis, negative regulation of cell proliferation and innate immune response. It is possible that the classes of genes affected by inhibition of Cdk9 [42] and those found in our study are involved in a cross-talk depending on the cell type and the transcriptional requirement in response to environmental signals, although more work is required to establish this link.

As discussed above, expression of Cyclin T2 is constitutive while Cyclin T1 protein expression is inducible depending on the activation or differentiation status of primary T-cells and macrophages, respectively [8,12]. In the present study, the number of genes down-regulated when Cyclin T1 is depleted is much more (631) than those down-regulated when Cyclin T2 is depleted (292). This could be representative of the transcriptional requirements of the cell. When the cells are in resting or undifferentiated stage, the transcriptional program and metabolism needs to be regulated to maintain homeostasis and this may be reflected in the nature of the genes down-regulated upon Cyclin T2 depletion which include negative regulation of metabolism and transcription. The relevance of constitutive expression of Cyclin T2 is also underscored in the regulation of kinase activity, signal transduction, energy reserve metabolism. Once the cells are activated or undergo a program of differentiation, the regulation of genes probably shifts to those under control of Cyclin T1. As the transcriptional and metabolic needs of the cells increase in response to activation or differentiation signals, the nature of the genes controlled by Cyclins change to active cellular metabolism, nucleic acid metabolism, apoptosis and cell growth. In this context, our results are in agreement with those of Kohoutek *et al. *[27] as they found that depletion of Cyclin T2 and not Cyclin T1 affected genes important for early embryogenesis while genes involved in cell communication was repressed when Cyclin T1 and not Cyclin T2 was depleted. We found that the number of genes affected by depletion of Cyclin T2 is lower than those affected by Cyclin T1 depletion. It is likely that the importance of multiple P-TEFb complexes in a cell lies probably not just in the number but the identity and function of the regulated genes. For instance, we found that Ankyrin repeat domain 12 was down-regulated when Cyclin T2 and not Cyclin T1 was depleted, while Ankyrin repeat domain 29 was repressed when Cyclin T1 and not Cyclin T2 was depleted. Ankyrin repeat domains mediate protein-protein interactions and are observed in bacterial and eukaryotic proteins [33,34]. In particular Ankyrin repeat domains have been reported to be present in IκB proteins and mediate interaction with NF-κB [35]. These domains are involved in important biological functions like transcriptional regulation, cell cycle and differentiation [36]. As discussed above, we note that there are both similarities and differences between the Kohoutek *et al.*[27] study in ES cells and our study in HeLa cells with respect to genes regulated by Cyclin T2 and Cyclin T1. It is likely that the differences arise because of experimental approaches. Despite these differences, the data presented in our study and by Kohoutek and colleagues should prove useful for researchers to mine for studies of specific genes or pathways of interest.

In summary, we have carried out transcriptional profiling to gain an insight into the genes regulated by the two distinct regulatory subunits of P-TEFb, Cyclin T2 and Cyclin T1. We found that there is limited redundancy in the genes under control of either Cyclins. The identity of the genes regulated by Cyclin T2 and T1 and the expression patterns of the Cyclin proteins are consistent with the notion that Cyclin T2 plays an important role in regulating genes involved in quiescent cells, while Cyclin T1 plays an important role in regulating genes in metabolically active cells.

## Methods

### Cell culture, Cell extracts and immunoblots

HeLa cells were purchased from American Type Culture Collection (ATCC) and were maintained in DMEM (Invitrogen) with 10% FBS, 100 units of penicillin, and 100 μg/ml streptomycin. Cell extracts were prepared by incubating cells in lysis buffer (50 mM Tris, 120 mM NaCl, 0.5% NP-40) containing protease inhibitors (2 μg/ml aprotinin, 1 μg/ml leupeptin, 2.5 mM phenylmethylsulfonyl fluoride) as described previously [11]. Protein concentrations were determined by a Bio-Rad protein assay, and 20 μg of total protein was loaded onto 10% SDS-PAGE gels. The procedure for immunoblots using enhanced chemiluminescence for detection has been previously described [43]. Antibody to β- actin was purchased from Sigma, and other antibodies were purchased from Santa Cruz Biotechnology.

### ShRNA design, Lentiviral production and Flow Cytometry

The target short hairpin RNA (shRNA) sequences used in this study were: shRNA CycT1: GCAGCGTCTTAACGTCTCA; shRNA -Cyc T2, GCCAGTACCTCTAA; shRNA -Control (MM), GCTATAGCTGTTCTAGTTC. The subcloning protocol into the FG12 self-inactivated lentiviral vector that carries an eGFP expression cassette has been described before [10]. The FG12 vector does not encode any viral gene products [44]. Briefly, oligonucleotides containing the target sequences with restriction enzyme site compatible overhangs were annealed and inserted into a hU6-1 plasmid vector immediately after the human U6 promoter. The shRNA expressing cassette was then subcloned into the FG12 vector. Stocks of the FG12 lentiviral vectors pseudotyped with vesicular stomatitis virus (VSV)-G were produced by either calcium phosphate mediated or Lipofectamine 2000 (Invitrogen) transient transfection of HEK-293T cells. Briefly, HEK-293T cells were cultured in 100-mm dishes in DMEM (Invitrogen) containing 10% FBS (Invitrogen), 100 units of penicillin, and 100 μg/ml streptomycin. The cells were cotransfected with 5 μg of each plasmid: vector plasmid, the VSV-G expression plasmid pHCMV-G, and the HIV-1 lentiviral packaging plasmids pRSV/REV and pMDLg/pRRE. Media was changed the following day and supernatants were passed through a 0.45-μm pore size sterile filter 48 hours post-transfection. Viral supernatants not used immediately were stored in aliquots at -80°C. HeLa cells were transduced at a multiplicity of infection (m.o.i.) of five in the presence of 5 ng/ml polybrene (Sigma). Transduction efficiencies were determined five days after lentiviral infection by suspending cells at 1 × 10^6 ^cells/ml in phosphate-buffered saline (PBS) with 2% FBS and the percentage of GFP positive cells were determined by flow cytometry using a Beckman-Coulter XL-MCL cytometer.

### MTT cell viability assay

HeLa cells transduced with lentiviral shRNA vectors against Cyclin T1, Cyclin T2 and Control (MM) were sorted for GFP expression using a flow cytometer. One hundred thousand sorted cells were plated and at different time points MTT was added at 5 mg/mL. After an incubation of 4 h, the formazon product was dissolved in acidified isopropanol and color estimated at 560 nm.

### Microarray analysis

HeLa cells were transduced with shRNA lentiviral vectors against Cyclin T2, Cyclin T1 or MM control as described above. Two independent biological replicate experiments were carried out. The cells were harvested five days post-transduction and total RNA for microarray analysis was extracted using Qiagen RNeasy Kit according to manufacturer's protocol and RNA quality was determined using an Aligent 2100 Bioanalyzer and the Nano-Drop ND-1000 Spectorphotometer. The RNA was reverse transcribed and the resultant cDNA transcribed using T7 RNA polymerase and biotinylated ribonucleotides to generate labeled cRNA. Fragmented cRNA was hybridized to U133 plus 2.0 human gene chips (Affymetrix) containing nearly 55,000 probe sets representing over 18,953 transcripts. Following washing and staining, the arrays were scanned using an Affymterix Gene Chip Scanner 3000, normalized to the medium intensity and analyzed. The primary microarray data have been deposited in the NCBI GEO database and are accessible through GEO Series accession number, GSE28339http://www.ncbi.nlm.nih.gov/geo/query/acc.cgi?acc=GSE28339. The microarray data was analyzed using the GeneSifter microarray data analysis system (VizX Labs LLC, Seattle, WA; http://www.genesifter.net). The program identifies differentially expressed genes and establishes the biological significance based on Gene Ontology (GO) http://www.geneontology.org and KEGG pathways http://www.genome.jp/kegg/pathway.html. The CEL files for each array were uploaded into GeneSifter and the data was normalized and log transformed using the GC-RMA algorithm. GC-RMA is the modified version of RMA (robust multi-array average) that uses probe sequence information for the background correction [45]. Differentially expressed genes were identified using Student t-test and a threshold of 1.2 was used to limit the data set to genes. Correction for multiple testing was performed using the Benjamini-Hochberg method to correct for false discovery rate.

The biological process ontologies and KEGG pathway terms associated with the differentially expressed genes were examined using a z-score report. The z-score report identifies ontologies or pathway terms that are significantly over-represented in a gene list [46]. Thus, a positive z-score indicates that more genes than expected beyond random chance fulfil the criteria (fold change and statistical criteria) in a certain group or pathway suggesting changes in that group or pathway. A negative z-score indicates that there were fewer genes than expected beyond random chance that met the fold change and statistic criteria. A functional annotation analysis was performed using DAVID http://david.abcc.ncifcrf.gov[47,48] to identify the link between diseases and the gen lists.

### Real-time PCR analysis

Microarray data were validated by quantitative real-time RT-PCR using the Bio-Rad MyIQ single color detection system as previously described [14]. Briefly, 1 μg of cellular RNA was reverse transcribed using the iScript cDNA synthesis kit (Bio-Rad). Quantitative real-time PCR was performed using the iQ SYBR Green Supermix (Bio-Rad) in the Bio-Rad iCycler. Primers for quantitative PCR were designed Beacon Designer 2.0 (Premier Biosoft). Primers used were: Cyclin T2 (forward) GGCGGAGGAGGAAGTGTCATG, Cyclin T2 (reverse) GCGGCTCGGCGTGTTCTC; Cyclin T1 (forward) AACCTTCGCCGCTGCCTTC, Cyclin T1 (reverse) ACCGTTTGTTGTTGTTCTTCCTCTC, HEXIM1 (forward) GCAGTTGGAAGTTGGCAGGTG, HEXIM1 (reverse) TCAGTTCTCCTCCGCCTCCTC; OAS1 (forward) GAGCCTCATCCGCCTAGTCAAG, OAS1 (reverse) CCCAAGCATAGACCGTCAGGAG; MFAP5 (forward) CCTGGCTTTCTTGCTCTCCCTC, MFAP5 (reverse) CGAGTCCTTTGGCTGCTGAATG; CDKN1C (forward) CGGACGAGACAGGCGAACC, CDKN1C (reverse) GCGGCGGCTACCTGACTG; CRM1 (forward) CCACCTTGATTCGTCCCCTCTC, CRM1 (reverse) ACCAACTGCTCCTTCCTTCCTC; GAPDH (forward) CGCCAGCCGAGCCACATC, (reverse) AATCCGTTGACTCCGACCTTCAC; β-Actin (forward) AGCAAGCAGGAGTATGACGAGTC, β-Actin (reverse) AGAAAGGGTGTAACGCAACTAAGTC. Analysis was performed using the MyIQ software program (Bio-Rad) and the fold changes were calculated using either β-Actin or GAPDH as a reference control as described earlier [49].

## List of abbreviations

RNAP II: RNA polymerase II; Cdk9: Cyclin dependent kinase 9; NELF: Negative elongation factor; DSIF: 5, 6-dichloro-1-β-D-ribofuranosylbenzimidazole; HEXIM1: hexamethylene bisacteamide-inducible 1.

## Competing interests

The authors declare that they have no competing interests.

## Authors' contributions

R.R. carried out experiments, analyzed the data and wrote the manuscript. W.Y. constructed the shRNA vectors. A.P.R. conceived of the study and wrote the paper. All authors read and approved the final manuscript.

## Authors' information

R.R. is a postdoctoral fellow and A.P.R. is Nancy Chang Professor in Department of Molecular Virology & Microbiology, Baylor College of Medicine, Houston, TX. W.Y. is currently a Resident in the Department of Pathology, University of Miami Miller School of Medicine.
